# The first case of primary hypertrophic osteoarthropathy with soft tissue giant tumors caused by *HPGD* loss-of-function mutation

**DOI:** 10.1530/EC-19-0149

**Published:** 2019-05-07

**Authors:** Qianqian Pang, Yuping Xu, Xuan Qi, Yan Jiang, Ou Wang, Mei Li, Xiaoping Xing, Ling Qin, Weibo Xia

**Affiliations:** 1 Department of Endocrinology, Key Laboratory of Endocrinology, Ministry of Health, Peking Union Medical College Hospital, Chinese Academy of Medical Sciences, Beijing, China; 2 Musculoskeletal Research Laboratory and Bone Quality and Health Assessment Centre, Department of Orthopedics & Traumatology, The Chinese University of Hong Kong, Hong Kong SAR, Hong Kong; 3 Department of Endocrinology, The First Affiliated Hospital of Shanxi Medical University, Taiyuan, Shanxi, China

**Keywords:** primary hypertrophic osteoarthropathy, PHO, HPGD mutation, soft tumor, COX2 selective inhibitor treatment

## Abstract

**Background:**

Primary hypertrophic osteoarthropathy (PHO) is a rare genetic multi-organic disease characterized by digital clubbing, periostosis and pachydermia. Two genes, *HPGD* and *SLCO2A1*, which encodes 15-hydroxyprostaglandin dehydrogenase (15-PGDH) and prostaglandin transporter (PGT), respectively, have been reported to be related to PHO. Deficiency of aforementioned two genes leads to failure of prostaglandin E2 (PGE2) degradation and thereby elevated levels of PGE2. PGE2 plays an important role in tumorigenesis. Studies revealed a tumor suppressor activity of 15-PGDH in tumors, such as lung, bladder and breast cancers. However, to date, no *HPGD*-mutated PHO patients presenting concomitant tumor has been documented. In the present study, we reported the first case of *HPGD*-mutated PHO patient with soft tissue giant tumors at lower legs and evaluated the efficacy of selective COX-2 inhibitor (etoricoxib) treatment in the patient.

**Methods:**

In this study, we summarized the clinical data, collected the serum and urine samples for biochemical test and analyzed the *HPGD* gene in our patient.

**Results:**

A common *HPGD* mutation c.310_311delCT was identified in the patient. In addition to typical clinical features (digital clubbing, periostosis and pachydermia), the patient demonstrated a new clinical manifestation, a giant soft tissue tumor on the left lower leg which has not been reported in *HPGD*-mutated PHO patient before. After 6-month treatment with etoricoxib, the patient showed decreased PGE2 levels and improved PHO-related symptoms. Though the soft tissue tumor persisted, it seemed to be controlled under the etoricoxib treatment.

**Conclusion:**

This finding expanded the clinical spectrum of PHO and provided unique insights into the *HPGD*-mutated PHO.

## Introduction

Primary hypertrophic osteoarthropathy (PHO; MIM 167100), also known as pachydermoperiostosis or idiopathic hypertrophic osteoarthropathy, is a rare genetic multi-organic disease characterized by digital clubbing, periostosis and pachydermia. Accompanying abnormalities include sebaceous hyperplasia, hyperhidrosis, acro-osteolysis, and effusions and pain of large joints (1, 2). Additional developmental anomalies, seen in a proportion of PHO patients, include patent ductus arteriosus and myelofibrosis (3, 4).

To date, two genes have been reported to be associated with PHO: hydroxyprostaglandin dehydrogenase (*HPGD*; MIM 601688), which encodes 15-hydroxyprostaglandin dehydrogenase (15-PGDH), and solute carrier organic anion transporter family, member 2A1 (*SLCO2A1*; MIM 601460), which encodes a prostaglandin transporter (1, 5). The pathogenesis and inherited pattern were controversial for a long time until the year 2008, and Uppal *et al.* identified the mutation of *HPGD* as the primary causative factor of PHO (1). Subsequently, Zhang *et al*. (5) confirmed the second gene *SLCO2A1* to be responsible for PHO. According to the molecular findings, PHO has been categorized into two subtypes: (1) hypertrophic osteoarthropathy, primary, autosomal recessive, type 1 (PHOAR1; MIM 259100), caused by *HPGD* deficiency and (2) hypertrophic osteoarthropathy, primary, autosomal recessive, type 2 (PHOAR2; MIM 614441), caused by *SLCO2A1* deficiency. Both HPGD and SLCO2A1 deficiency can independently lead to failure of PGE_2_ degradation, resulting in elevated levels of prostaglandin E2 (PGE_2_) in the circulation, which is thought to contribute to the pathogenesis for PHO (1, 6). PHO is a clinically heterogeneous disease. The onset age of PHO is bimodal distribution. Peaking onset age of clinical manifestations is usually the first year of life in PHOAR1 with *HPGD* mutations, and at puberty in PHOAR2 with *SLCO2A1* mutations (6). Sefiert *et al*. (7) revealed that manifestations of bones and joints found in patients with homozygous mutations in the *HPGD*, usually appear earlier than those in the *SLCO2A1*, suggesting the clinical heterogeneity between the two subtypes of PHO.

It is widely acknowledged that PGE_2_ plays an important role in the development of tumors. PGE_2_ can stimulate cell proliferation, angiogenesis and motility while inhibiting apoptosis and immune surveillance (8, 9, 10). Reduced 15-PGDH and SLCO2A1 are believed to contribute to elevated levels of PGE_2_ in the circulation thereby leading to the pathogenesis of these tumors (11, 12, 13, 14). However, in humans, reports of *HPGD* and *SLCO2A1* mutation cases have only been focused on the typical features such as digital clubbing, periostosis and pachydermia. Till 2014, Guda *et al.* (15) reported a French-Canadian family with *SLCO2A1* mutation presenting digital clubbing and early-onset colon neoplasm, suggesting a link between PHO and tumors. 15-PGDH is the major enzyme responsible for prostaglandin degradation. Numerous studies have demonstrated a tumor suppressor activity of 15-PGDH in a number of different tumors, such as lung, bladder and breast cancer (16, 17, 18). Whereas, to date, no *HPGD*-mutated PHO patients presenting concomitant tumor have been documented.

Up to now, no standard treatment has been approved for PHO due to the small number of patients with PHO at most clinical centers. There are some case reports with varying therapeutic option and response, and the treatments are mostly focused on alleviation of symptoms, including nonsteroidal anti-inflammatory drugs (NSAIDS), pamidronate and tamoxifen citrate to relieve painful osteoarthropathy (19, 20). Given that the increased circulating PGE_2_ levels is responsible for pathogenic mechanism of PHO, cyclo-oxygenase (COX) inhibition may represent a targeted therapeutic option. Recently, COX-2 selective inhibitors, which inhibit the COX-2 enzyme and thereby suppress PGE_2_ biosynthesis, represent promising treatment options for PHO. A few studies have shown that etoricoxib, a novel COX-2 selective inhibitor, has a positive therapeutic effect on PHO patients in terms of decreased urinary PGE_2_ levels and improvement of clinical phenotypes including pachydermia, clubbing finger and joint swelling (21, 22, 23).

Here we reported the first case of a Chinese *HPGD*-mutated PHO patient with soft tissue giant tumors at bilateral lower legs, as well as evaluated the efficacy of selective COX-2 inhibition (etoricoxib) treatment in this patient.

## Methods and materials

### Human subjects

This study was approved by the Local Ethics Committee of the Department of Scientific Research at Peking Union Medical College Hospital (PUMCH). The Chinese PHO patient signed informed consent documents before entering the study.

### Biochemical parameters

The patient was admitted into our hospital and went through detailed clinical, biochemical and radiographic investigation. For biochemical analysis, fasting blood samples and 24-h urine were collected. Serum calcium (Ca), serum phosphate (Pi), serum alkaline phosphatase (ALP), serum creatinine (SCr), erythrocyte sedimentation rate (ESR), hypersensitive C reactive protein (hsCRP), interleukin 6 (IL-6) and tumor necrosis factor-α (TNF-α) were measured spectrophostometrically using routine assays available at the central laboratory of PUMCH. Serum intact parathyroid hormone (iPTH) and beta- C-terminal telopeptide of type I collagen (β-CTX) were measured by an automated Roche electrochemiluminescence system (Roche Diagnostics). Serum and urinary PGE_2_ and PGEM (a metabolite of PGE_2_) levels were measured by competitive enzyme-linked immunosorbent assay (ELISA) according to the manufacturer’s instructions (Cayman Chemicals). The measuring ranges of ESR, hsCRP, IL-6, TNF-α, PTH, β-CTX, PGE_2_ and PGEM were 1–140 mm/h, 0.15–20 mg/L, 2–1000 pg/mL, 1.7–1000 pg/mL, 1.2–5000 pg/mL, 0.01–6.00 ng/mL, 7.8–1000 pg/mL and 0.39–50 pg/mL, respectively. The intra-assay coefficients of variation were <5% for ESR, 0.7% for hsCRP, 3.5–6.2% for IL-6, 2.6–3.6% for TNF-α, 1.2% for iPTH, 2.0% for β-CTX, 3.7% for PGE_2_ and 5.5% for PGEM.

### Imaging techniques

X-ray radiography of both hands and legs were performed to assess bony deformities. Magnetic resonance imaging (MRI) of the bilateral legs was performed to confirm the X-ray findings and evaluate the giant tumors at patient’s lower legs. Computed tomographic angiography (CTA, SOMATON Force, Siemens Healthineers) was conducted at the tumor sites to clarify the tumor source and vascular perfusion.

### Area bone mineral densities (BMD)

Area bone mineral densities of lumbar spine and proximal femur were measured with a dual energy X-ray absorptiometry (Prodigy Advance, GE Lunar Corporation).

### Mutational analysis

Whole blood was obtained from the patient. Genomic DNA was extracted from peripheral white blood cells using the DNA Extraction Kit (QIAamp DNA, Qiagen) according to the manufacturer’s instructions. The seven exons of *HPGD* were amplified through PCR with a set of primers designed by Gene Runner Primer Analysis Software. The amplified products were sequenced by an automated sequencer (ABI 373XL sequencer, Applied Biosystems) according to the manufacturer’s recommendation. Putative mutations were analyzed and compared using the Basic Local Alignment Search Tool (Blast).

### Bioinformatics analysis

The identified mutation in *HPGD* gene was analyzed at the protein level. Protein modeling was conducted based on the data of 15-PGDH structure in Protein Date Bank (PDB ID: 2GDZ, http://www.rcsb.org), and the mutational-related residues were positioned in the constructed 3D structural model (24) using the PyMOL Viewer 1.8.6 (free download from https://pymolwiki.org).

## Results

### Clinical findings

**Figure 1:**
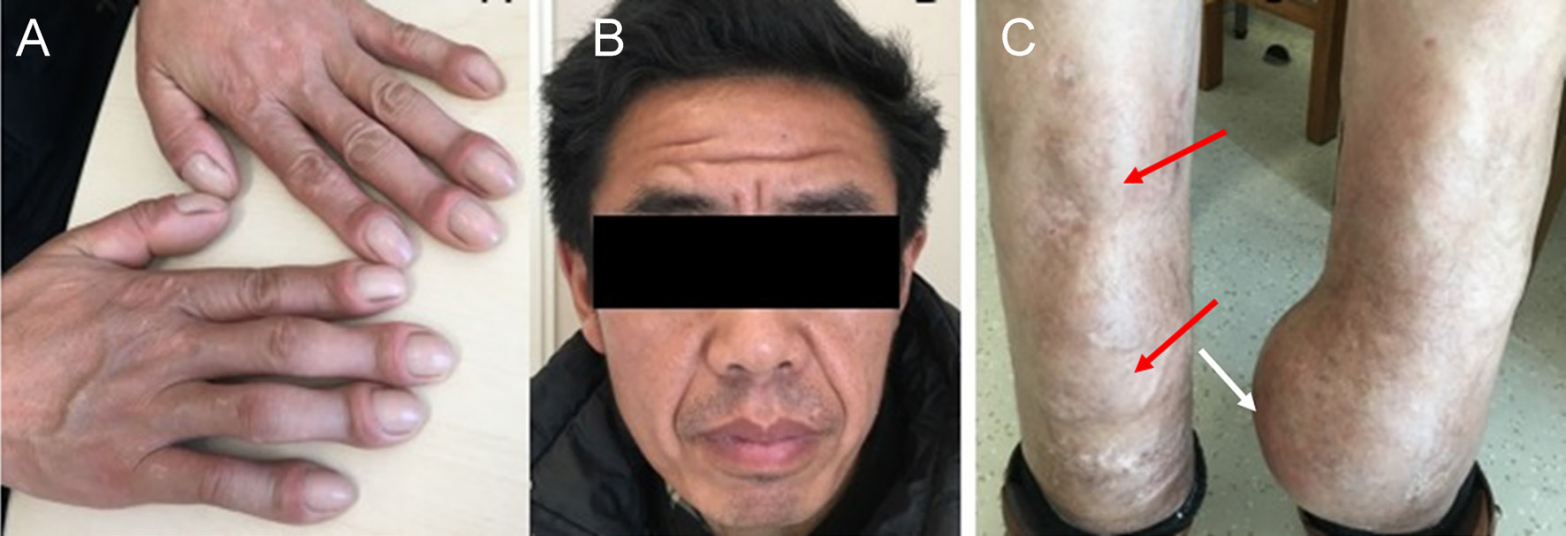
The clinical features of the *HPGD*-mutated PHO patient. (A). Digital clubbing. (B) Oily, thickened and furrowed face. (C) A soft tissue giant tumor on the left lower leg (white arrow), two smaller tumors on the right leg (red arrow).

**Figure 2:**
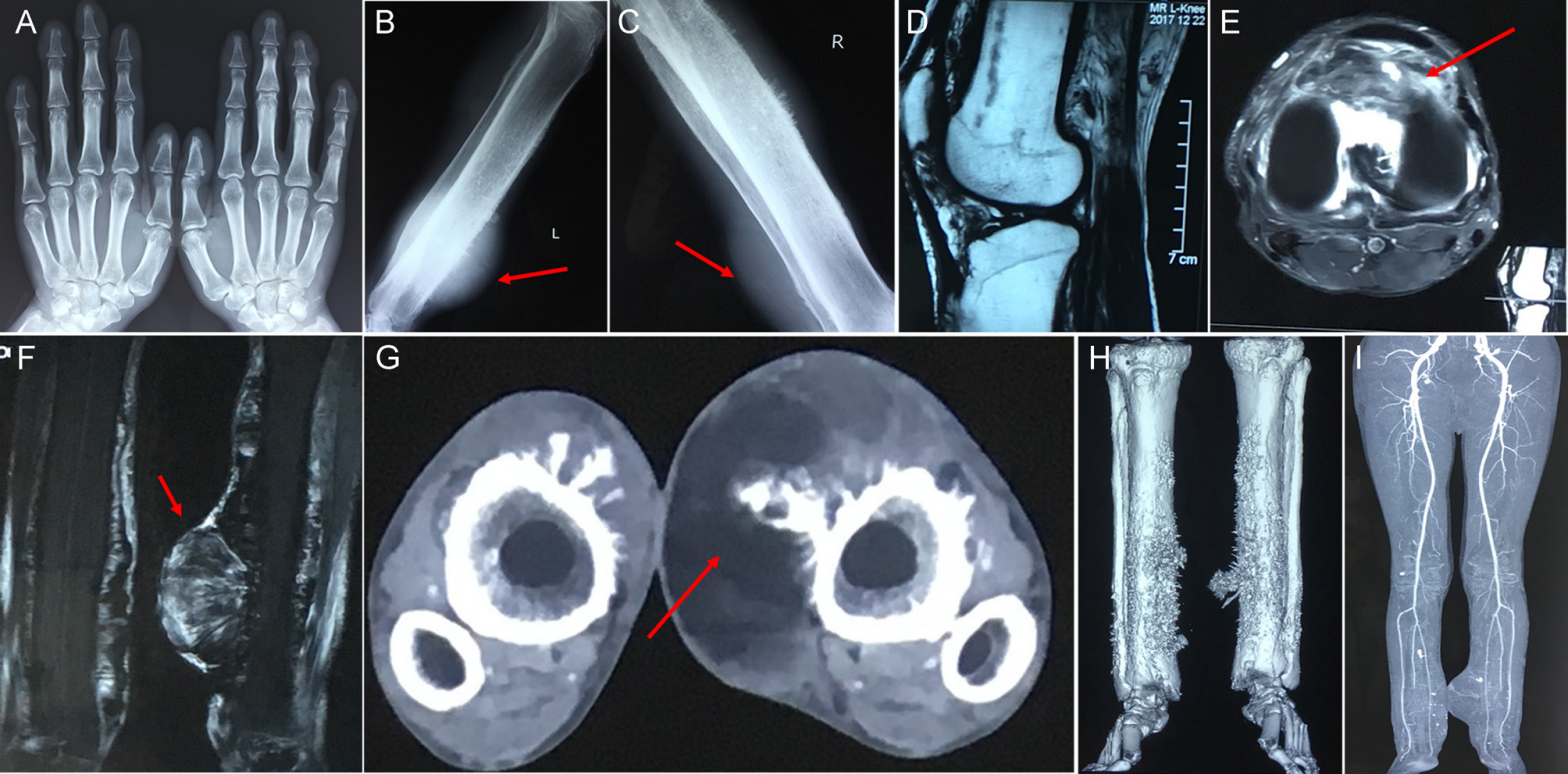
The radiographic signatures of the *HPGD*-mutated PHO patient. (A and C) X-ray examination of hands and bilateral knees. (A) Acro-osteolysis on distal phalanges of the fingers. (B and C) Cortical thickening and periostosis in the long bones, as well as the soft tissue swelling on the lower legs (red arrows). (D and E) MRI examination of bilateral legs. (D) Thickening of cortical bones and bone marrow edema in femurs and tibias. (E) Diffuse synovial hypertrophy and effusion in knee joint (red arrow). (F) An extra-articular diffuse tumor of the medialis of left tibia (6.2 × 11.2 × 10.6 cm) (red arrow). (G and H) CTA examination of bilateral legs. (G) Calcified soft tissue mass in the legs bilaterally, the biggest was in the left (11.3 × 5.5 cm) (red arrow). (I) Normal vascular perfusion of bilateral legs. And no increased vascularity was found inside the giant tumor.

**Figure 3:**
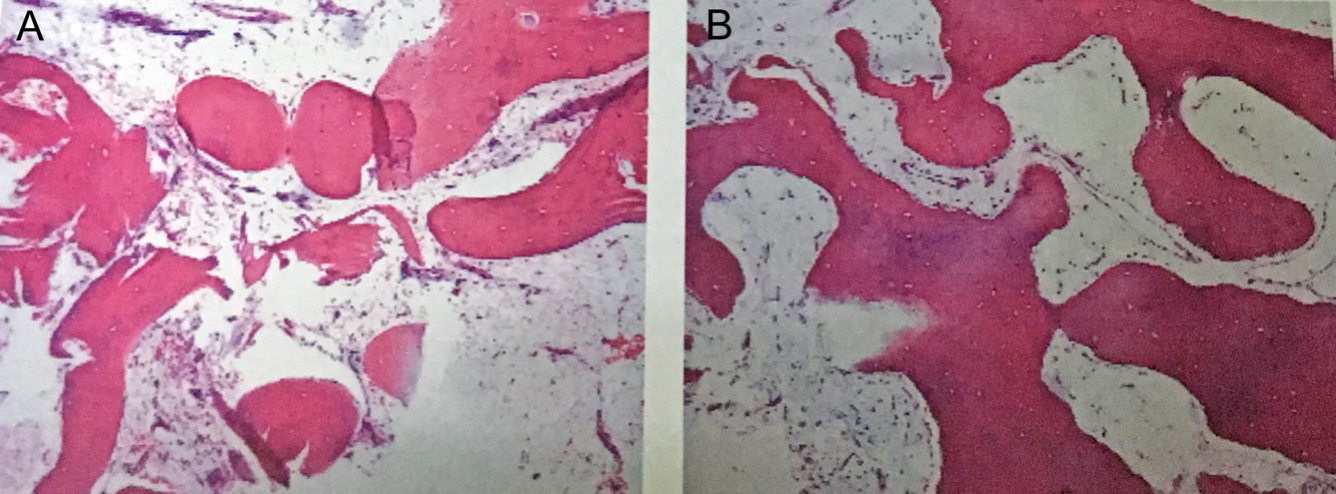
Histological images analysis. Photomicrograph of the tissue tumor obtained from the left leg (A and B) showing irregularly curved immature woven bone trabeculae with more dense proliferative fibrous granulation tissues invading among the trabecular bones. Soft tissue ossification was also found in this tissue but without evidence of a malignant neoplasm (HE staining: 0×).

The 41-year-old patient was born to healthy consanguineous parents. Widening of distal phalanges of fingers, hyperhidrosis of hands and facial furrowing were noted during infancy. He complained of frequent pain in bilateral knees after having a cold. From the age of 35 years, he had swelling in knees and ankles but denied any bone pain. One year later, he noticed a soft tumor at his left leg, and the size of the tumor increased rapidly in the following years. At the age of 41 years, he was admitted to our clinic with complains of a giant tumor at left leg. Physical examination showed digital clubbing (Fig. 1A), oily, thickened and furrowed face (Fig. 1B), palmoplantar hyperhidrosis and palmoplantar hyperkeratosis. Swelling was found in bilateral wrists and knees (circumference of left and right knee was 37.0 cm and 38.0 cm, respectively). He suffered from a total of three soft tumors at bilateral legs, and the most giant one located at left lower leg (10 × 12 cm, circumference 44.5 cm), and the other two smaller tumors located at right lower leg (Fig. 1C). He had no cardiac, pulmonary, hepatic disease, as well as delayed closure of cranial suture, anemia or hypoalbuminemia. He denied any gastrointestinal discomfort. The laboratory findings were shown in Table 1. Area bone density of lumbar spine and proximal femur were in normal range. Radiological examination of both hands and legs showed acro-osteolysis on distal phalanges of fingers (Fig. 2A) and periostosis along long bones (Fig. 2B and C). Besides, X-ray of bilateral legs revealed massive soft tissue swelling of calf (Fig. 2B and C). MRI of legs confirmed the plain radiographic findings and also showed subchondral cysts, diffuse synovial hypertrophy and effusion in bilateral knees (Fig. 2D and E). Additionally, MRI imaging showed round hyperdense foci around medial of the midshaft of the left tibia (6.2 × 11.2 × 10.6 cm), as well as round hyperdense foci at the right upper fibular (1.0 × 1.2 cm) (Fig. 2F). CTA revealed the calcified soft tissue mass at medialis of bilateral legs. The biggest one was at left (11.3 × 5.5 cm), indicating that the tumor was soft tissue source (Fig. 2G and H). Vascular perfusion of bilateral legs was normal (Fig. 2I). The patient conducted tumor biopsies in local hospital (The Third Hospital of Hebei Medical University) before he was admitted to our department for medical consulting. Histological evaluation of the tumor tissue revealed a benign tumor with irregularly curved immature woven bone trabeculae and proliferative fibrous granulation tissues invading among the trabecular bones (Fig. 3). PHO diagnosis was made by typical features including digital clubbing, hyperhidrosis, periostosis and acro-osteolysis, as well as the increased levels of serum PGE_2_. The patient has two siblings, and all of his relatives were healthy without any typical PHO symptoms.

**Table 1:** Laboratory findings of the PHO patient before and after etoricoxib treatment.

	Before treatment	After treatment	Reference range
Routine biochemical markers			
ALT (U/L)	44	16	9–50
SCr (µmol/L)	68	62	59–104
Ca (mmol/L)	2.22	2.17	2.13–2.70
Pi (mmol/L)	1.16	1.02	0.81–1.45
ALP (U/L)	**130**	102	45–125
iPTH (pg/mL)	56.3	47.9	12–65
β-CTX (ng/mL)	**0.827**	**0.654**	0.26–0.512
Inflammatory cytokines			
ESR (mm/h)	6	NA	0–15
hsCRP (mg/L)	**33.4**	0.78	0–3
IL-6 (pg/mL)	**9.0**	2.0	<5.9
TNF-α (pg/mL)	4.7	4.8	<8.1
Specific biochemical markers			
Serum PGE2 (pg/mL)	**316**	231	28.7–308.2 (26)
Serum PGEM (pg/mL)	13.3	**24.1**	0.5–17.2 (26)
Urinary PGE2 (ng/mmol cr)	**618**	**215**	36.4–85.5 (25)
Urinary PGEM (ng/mmol cr)	**4.27**	**3.27**	23–52.8 (25)

Abnormal findings were indicated in bold.

ALP, serum alkaline phosphatase; ALT, alanine aminotransferase; β-CTX, beta-C-terminal telopeptide of type I collagen; Ca, calcium; ESR, erythrocyte sedimentation rate; hsCRP, hypersensitive C reactive protein; IL-6, interleukin 6; NA, not available; Pi, phosphate; PTH, parathyroid hormone; Scr, serum creatinine; TNF-α, tumor necrosis factor-α.

### The treatment of patient

The patient was treated with selective COX-2 inhibitor (etoricoxib, 60 mg once daily, Merk Sharp & Dohme Corp USA) and re-evaluated at the time point of 6 months. After re-evaluation, we found etoricoxib treatment led to the reduction of swelling in knees (circumference decreased from 38 to 37.1 cm) and ankles than it was before etoricoxib treatment, though facial furrowing appearance persisted. The soft tissue mass in bilateral legs persisted but did not evolve since etoricoxib treatment. Serum and urinary PGE_2_ levels, inflammatory cytokines and bone turnover markers all decreased after treatment. The levels of the biochemical markers before and after treatment were shown in Table 1. MRI of the bilateral knees showed remission of synovitis.

### Mutational analysis of *HPGD*

**Figure 4:**
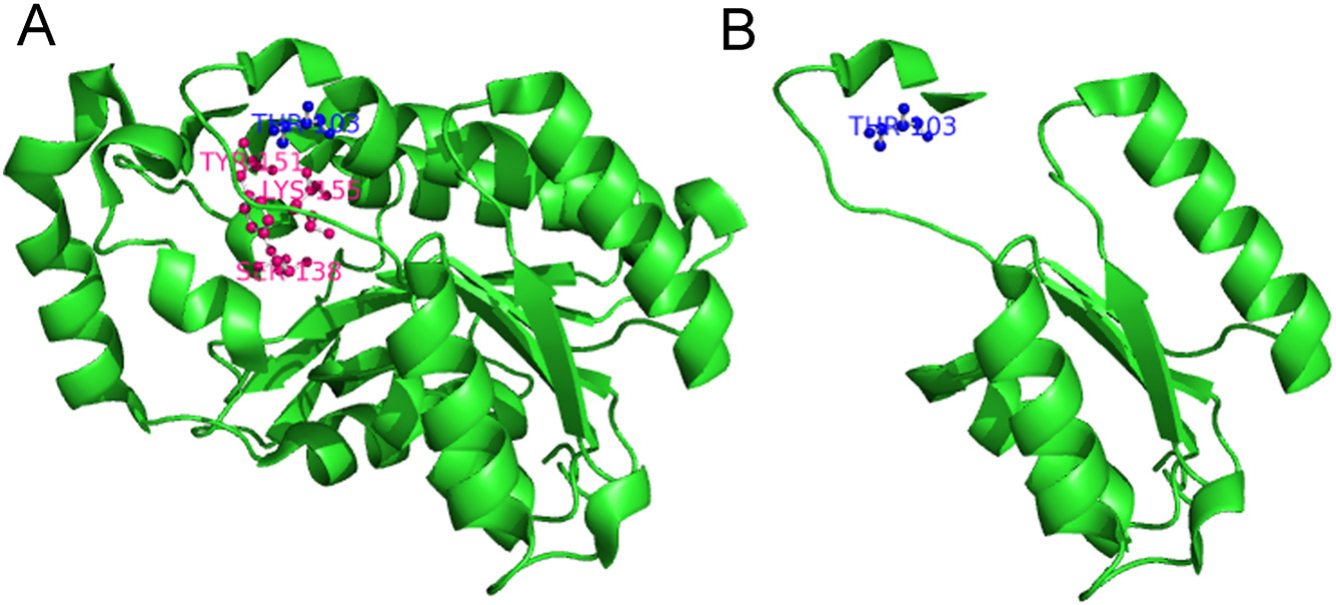
The 3D structure of human wide type and mutated 15-PGDH protein. (A) The 3D structure of human 15-PGDH protein was constructed by Niesen *et al*. (24), in which the deletion mutation-related residues (103T) and active sites Ser138, Tyr151 and Lys155 were labeled blue and violet, respectively. (B) The 3D structure of mutated 15-PGDH protein. The deletion c.310_311delCT mutation caused the frameshift after 103T and a premature stop at codon 106, leading to a truncated protein and 160 amino acids lacking.

Direct sequencing of genomic DNA indicated that the patient carried a homozygous mutation c.310_311delCT. This mutation caused frameshift after 103T and created a premature TAA stop signal at codon 106 which resulted in a truncated protein. The mutation-related residues (103T) and predicted truncated protein of *HPGD* gene were shown in Fig. 4. The deletion c.310_311delCT has been reported in some Asian PHO families (19, 22, 25), which seems to be a hotspot mutation in Asian PHO patients.

## Discussion

PHO is a rare genetic disease featured by digital clubbing, periostosis, pachydermia and acro-osteolysis, which has been linked to the failure of prostaglandin metabolism. Defects of two genes have been confirmed to be responsible for this disease: *HPGD* and *SLCO2A1*, which encodes 15-PGDH and prostaglandin transporter (PGT), respectively (1, 5). Under normal conditions, PGE_2_ is metabolized and cleared through two main steps: (1) selective uptake of PGE_2_ across the plasma membrane by PGTs, including SLCO2A1, SLCO3A1, SLCO4A1. (2) degradation of PGE_2_ inside the cell by 15-PGDH into PGEM (26). 15-PGDH is the main enzyme in prostaglandin metabolism. Patients with *HPGD* loss-of-function mutations present elevated PGE_2_ levels (1, 27). Consistently, in the current study, our patient showed an increased level of serum and urinary PGE_2_ (Table 1).

To date, *HPGD* mutations have been reported in 47 families, of which 20 were from Southern or Western Asia (1, 3, 19, 27, 28, 29, 30). In the present study, a previously known *HPGD* mutation (c.310_311delCT) was identified in our patient. The mutation c.310_311delCT, which contributed to a frameshift after codon 104 and resulted in the loss of protein acceptor site as well as the putative substrate-binding site, is currently considered to be the most common mutation in Asian familial cases (19). In Yuan’s study, this common mutation was found in all the nine reported Chinese patients, indicating the high frequency of c.310_311delCT mutation in Chinese PHO patients (25).

The *HPGD* gene encodes a helical protein, consists of 266 amino acids, which belongs to the member of the short-chain dehydrogenase family (SDR) family (31). Generally, SDRs are divided into two larger families, ‘classical’ with 250-odd residues and ‘extended’ with 350-odd residues. 15-PGDH belongs to the ‘classical’ SDR family. Specifically, the structure of 15-PGDH (the ‘classical’ SDR enzyme) includes N-terminal transmembrane domains, C-terminal transmembrane domains, nucleotide-binding region, active site, central β-sheet and the helix. Previous studies have revealed that the active sites Ser138, Tyr151 and Lys155 are the most conserved residues essential for catalytic activity of 15-PGDH. Tyr151 functions as the catalytic base, whereas Ser138 stabilizes the substrates, and Lys155 forms hydrogen bonds with the nicotinamide ribose moiety to promote proton transfer (32, 33). Niesen *et al.* (24) have constructed a 3D structure of human 15-PGDH by homology modeling technique. The deletion mutation c.310_311delCT encodes a truncated protein lacking 160 amino acids of the C-terminal domain, which might interfere the stability and function of 15-PGDH structure. Ultimately, this mutation would result in loss of function of the enzyme since the most important residues (Ser 138, Tyr151 and Lys155) were all absent from the protein (Fig. 4). In view of the fact that 15-PGDH is the main enzyme in prostaglandin metabolism, the alteration of the 15-PGDH structure may be the cause of the variations in clinical phenotypes in PHO patients.

We reviewed all 51 *HPGD* mutant patients (PHOAR1) reported before and found almost all of these PHOAR1 patients have digital clubbing (50/51, 98%) and periostosis (51/51, 100%). Besides, accompanying abnormalities such as hyperhidrosis (41/51, 80.4%), joint swelling (30/51, 58.8%), various forms of pachydermia (28/51, 54.9%) and arthralgia (22/51, 43.1%) have also been seen in a proportion of the PHOAR1 patients (1, 21, 22, 23, 25, 29, 34, 35, 36). Consistent with Hou’s study (27), gastrointestinal complications, peptic ulcer, chronic gastritis, anemia and myelofibrosis which were only presented in PHOAR2 patients, were absent in these PHOAR1 patients. In keeping with previous findings of the reported *HPGD*-mutated patients, the dermatoskeletal features of the patient in this study were typical for PHOAR1. The patient presented an early-onset age in infancy, which was similar to those extensively reported in previous *HPGD*-mutated PHO patients (6). Additionally, the patient had typical phenotypes of digital clubbing, periostosis, pachydermia, hyperhidrosis and arthropathy but denied any gastrointestinal complications, peptic ulcer, chronic gastritis, anemias and myelofibrosis syndromes.

It was well known that PGE_2_ could stimulate the activity of both osteoclasts and osteoblasts, causing the enhancement of bone resorption and new bone formation, which might be related to PHO skeletal manifestations such as acro-osteolysis and periostosis (1). Jajic *et al.* (37) reviewed all of the reported 76 PHO patients and found all of these patients had periosteal reaction along the long bones and 23.7% of these patients had acro-osteolysis. Because the levels of bone turnover markers were not investigated in these patients, the association between PGE_2_ and bone homeostasis were still unknown in PHO patients. In our study, the patient showed acro-osteolysis at distal phalanges of the fingers (Fig. 2A) and periostosis at the shafts of tubular bones (Fig. 2B and C). Biochemical test of the patient showed elevated levels of β-CTX and ALT. Skeletal X-ray findings in combination with the increased levels of bone turnover markers, suggested that though area bone mineral densities (aBMD) were within the normal range, there might be still some skeletal changes independent of aBMD measured by DXA in PHO patients.

As discussed earlier, nearly half of PHOAR1 patients presented osteoarticular manifestations, including joint swelling (30/51, 58.8%) and arthralgia (22/51, 43.1%), but joint impairment assessment through radiographic imaging of these patients was lacking. Literature review provides MRI examination results in six PHO patients, but the regions of interest (ROI) of MRI were focused on the bones rather than the joints. Pineda *et al.* (38) demonstrated remodeling and thickening of cortical bone and tortuous intraosseous vascular channels. Adams *et al.* (34) found transverse long bone expansion with periosteal thickening using gadolinium-enhanced MR images. In the present study, MRI of bilateral legs was performed in the PHO patient. Both bones and joints were scanned and evaluated in the MRI examination and the results showed thickening of cortical bones and bone marrow edema in femurs and tibias (Fig. 2D). Moreover, joint swelling, subchondral cysts, diffuse synovial hypertrophy and effusion were also seen in knee joint (Fig. 2E), suggesting joint impairment of the patient.

It is worth mentioning that the patient had soft tissue tumors at bilateral lower legs, which has not been reported in PHOAR1 patients before. Studies have revealed that 15-PGDH expression was reduced in colon, breast, gastric and lung cancers and restoration of 15-PGDH could inhibit tumorigenesis in xenografts, indicating that 15-PGDH was a tumor suppressor in these tumors as well as played an essential role in regulating tumor development and progression (16, 17, 18). A work from Rogenski’s laboratory (17) showed that 15-PGDH knockdown in 9027 shRNA lentivirus-infected RT4 cells permitted PGE_2_ signaling as measured by cAMP generation, whereas signaling was suppressed in the 15-PGDH-ex-pressing parental lines, demonstrating that 15-PGDH had a direct impact on PGE_2_ signaling in cancer cells. Numerous studies have already revealed an important link between elevated levels of PGE_2_ and tumor development and progression, suggesting there was a potential relationship between PHO and tumors (8, 9, 10). However, up to now, there was only one *SLCO2A1*-mutated patient reported to suffer from colon neoplasm (15). In this study, we reported the first case of *HPGD*-mutated PHO patient, presenting typical PHO features and atypical soft tissue tumors at bilateral lower legs. It was well known that the effects of PGE_2_ were mediated via four known receptors EP1, EP2, EP3 and EP4 in individual target cell, involving in cell proliferation, apoptosis and angiogenesis (39). EP-related signaling seemed to play a key role in tumorigenesis. Most recently, via using mPGES1-deficient mice, PTGER4-deficient mice and specific antagonists of EPs, a study from Inada’s laboratory (40) assessing the role of PGE_2_ in the soft tissue tumors revealed that PGE_2_ acted on fibroblasts in tumor microenvironment through EP4 receptor. Besides, this research group also demonstrated that tumor growth and vascularization in soft tissues were abrogated by an EP4 receptor antagonist, suggesting PGE_2_/EP4 signaling played a critical role in the growth of tumors. Unfortunately, since the patient refused tumor biopsies in our department, the *in vitro* experiment was not available in this study. Whether PGE_2_/EP4 signaling involved and played a critical role in the tumorigenesis of this PHO patient remained unclear. Notably, radiographic examinations, especially CTA of lower legs, suggested benign soft tissue tumors as there was no increased vascularity and abnormal vascular perfusion found inside the giant tumor (Fig. 2I).

Studies concerning the treatment of PHO patients were limited and no treatment has been shown to reverse the hypertrophic bone changes. Currently, the most commonly used drugs were NSAIDS, since they were effective in alleviating arthritis and easing bone pain. Recently, the novel selective COX-2 inhibitors, etoricoxib, which not only suppress COX-2-derived PGE_2_ synthesis and lower PGE_2_ levels from upstream, but also improve biochemical selectivity over that of other selective COX-2 inhibitors (41, 42), has been shown to have a positive therapeutic effect on PHO patients in some clinical studies (21, 23). Indeed, the serum and urinary PGE_2_ in our patient were decreased after 6-month etoricoxib treatment, confirming its efficacy to treat this disease based on pathogenic considerations. The PHO-related symptoms including joint swelling and hyperhidrosis, as well as the increased biochemical markers including ALP, β-CTX, hsCRP and IL-6 have also been markedly improved within 6 months. These findings were in agreement with the Li and Yuan’s study (21, 23). Interestingly, though the soft tissue tumor persisted, it has not grown since COX-2 inhibitor treatment, indicating the PGE_2_ might play a central role in tumor progression of PHO patient.

In summary, in the present study, we reported for the first time a Chinese Han PHO patient with soft tissue giant tumors in lower legs. Mutational analysis of the patient revealed a common homozygous mutation c.310_311delCT in *HPGD* gene. In addition to typical clinical features including digital clubbing, periostosis, pachydermia and acro-osteolysis, the patient presented an atypical manifestation, giant soft tissue tumors at both lower legs which have not been reported in *HPGD*-mutated PHO patient before. After six-month treatment with a novel selective COX-2 inhibitor, etoricoxib (60 mg once daily), PGE_2_ levels were markedly decreased and PHO-related symptoms such as pachydermia, joint swelling and hyperhidrosis significantly improved in this patient. Though the soft tissue tumors persisted, it seemed to be controlled under the etoricoxib treatment. Further studies and investigations should be performed to reveal the role of PGE_2_ signaling on tumorigenesis in PHO.

## Declaration of interest

The authors declare that there is no conflict of interest that could be perceived as prejudicing the impartiality of the research reported.

## Funding

This study was supported by the National Natural Science Foundation of China (Grant number: 81471088 and 81670714).
